# American Dental Association

**Published:** 1886-09

**Authors:** 


### American Dental Association.

The annual meeting of this body was held on the first Tuesday and the three succeeding days of August.

In many respects it was an admirable meeting, many valuable papers were presented and discussed. Almost every section had something of general interest; many new and valuable instruments and appliances were presented for the inspection of the members.

No member of our profession can attend such a meeting without coming away better qualified for the performance of his work.

Professional politics, which has been more or less prevalent at the meetings of this body for a number of years seemed to culminate on this occasion, and it is to be hoped that everybody became so thoroughly disgusted with that sort of business, that hereafter, it will be excluded.

Political maneuverings have been a great embarrassment and obstacle to the efficient work of the Society for several years. It is not our purpose here to censure anybody for this state of affairs. We will only say, that it is an easy matter for parties to be formed, and factions to arise, unless care is exercised.

It is to be hoped that every member of the Association will from this henceforth resolve that the experience of the past shall suffice for this kind of demonstration.

The American Dental Association has been the representative body of the profession in this country, and has been the means of great good. We anticipate for it a brighter career.

Let every true member of the profession exercise his best efforts for this result.

This number of the REGISTER contains a report of the papers, discussions, and proceedings of the entire meeting.

